# Facilitated Structure Formation in Isoporous Block Copolymer Membranes upon Controlled Evaporation by Gas Flow

**DOI:** 10.3390/membranes10050083

**Published:** 2020-04-28

**Authors:** Kirti Sankhala, Joachim Koll, Volker Abetz

**Affiliations:** 1 Helmholtz-Zentrum Geesthacht, Institute of Polymer Research, Max-Planck-Strasse 1, 21502 Geesthacht, Germany; 2 Institute of Physical Chemistry, Universität Hamburg, Martin-Luther-King-Platz 6, 20146 Hamburg, Germany

**Keywords:** isoporous membranes, PS-*b*-P4VP diblock copolymer, gas flow, evaporation-induced self-assembly, gSNIPS, SNIPS, microphase separation

## Abstract

The conventional fabrication of isoporous membranes via the evaporation-induced self-assembly of block copolymers in combination with non-solvent induced phase separation (SNIPS) is achieved under certain environmental conditions. In this study, we report a modification in the conventional fabrication process of (isoporous) flat sheet membranes in which the self-assembly of block copolymers is achieved by providing controlled evaporation conditions using gas flow and the process is introduced as gSNIPS. This fabrication approach can not only trigger and control the microphase separation but also provides isoporous structure formation in a much broader range of solution concentrations and casting parameters, as compared to fabrication under ambient, uncontrolled conditions. We systematically investigated the structure formation of the fabrication of integral asymmetric isoporous membranes by gSNIPS. A quantitative correlation between the evaporation conditions (causing solvent evaporation and temperature drop) and the self-assembly of block copolymers beginning from the top layer up to a certain depth, orientation of pores in the top layer and the substructure morphology has been discussed empirically.

## New Concept

The isoporous block copolymer membranes fabricated via the SNIPS (self-assembly of block copolymers in combination with non-solvent induced phase separation) process were invented more than a decade ago. By providing uniform pores on an integrally asymmetric porous structure, the membranes promise to be an outstanding candidate for efficient separation processes. However, the delicate phenomenon of self-assembly of block copolymers requires specific conditions in a narrow window of parameters which challenges their reproducibility, with any change in the block copolymer solution, the temperature and/or humidity thus constraining upscaling. Herein, we report a way to achieve the self-assembly of block copolymers by controlled evaporation using a controlled gas flow, which results in a controlled development of the concentration gradient from the top surface into the solution film and thus helps to direct the microphase separation. The gas flow induced self-assembly of block copolymers in combination with non-solvent induced phase separation is introduced as “gSNIPS”. By gSNIPS, we could widen the window of parameters for the desired structure formation of the membranes, offering a favorable effect on the production and optimization of the processing parameters. The controlled evaporation and fast (within 2 s) development of self-assembled pores on the top surface can reduce the production cost. 

## 1. Introduction

Block copolymers, their self-assembly and the self-assembled structures have been researched categorically in the last decades, exploring their vast potential in various fields [1,2,3,4,5,6,7,8,9,10]. One of the potential applications of block copolymers is in the fabrication of isoporous membranes, providing a high density of uniform functional pores (typically > 10^14^ pores/m^2^) on the top surface along with a tunable diameter and the perpendicular orientation above a macroporous asymmetric substructure support [11,12,13,14,15,16,17,18,19,20,21,22]. In the conventional fabrication method of isoporous block copolymer membranes, a homogenous block copolymer solution is either cast or spun in order to fabricate flat sheet or hollow fiber membranes, respectively [23,24,25]. Simultaneously, the exposure to the environment for a period of tens of seconds allows evaporation-induced microphase separation of block copolymers via nucleation and growth. In this way, the desired morphology during the non-equilibrium self-assembly of block copolymers is found only in a narrow range of parameters and that must be fixed by non-solvent induced phase separation (NIPS); the overall process of self-assembly of block copolymers followed by NIPS is abbreviated as the SNIPS process [23]. The complex parameter landscape associated with the isoporous membranes demands a fine-tuning of fabrication parameters. The parameters include the chemical composition and molar mass of block copolymer, the concentration and solvent composition of the casting solution, the environmental conditions and the evaporation time (*t*) to trap the desired pore structure [26,27]. The study of isoporous membrane fabrication using polystyrene-*block*-poly(2-vinylpyridine) (PS-*b*-P2VP) diblock copolymer showed that the critical conditions required for isoporous structure formation exist in a narrow window of solution composition and fabrication conditions, various transient morphologies could be seen in the path to achieving a regular pore network with the further possibility of structural changes [28]. Although this is comparatively less complex in the case of highly segregated polystyrene-*block*-poly(4-vinylpyridine) (PS-*b*-P4VP) diblock copolymer, the identification of the optimal parameters for each block copolymer, each having different composition and molecular weight demands, necessitates considerable trial and error efforts that hinder progress in this field [26]. 

Experimentally, many approaches like blending or additives in the casting solution [29,30,31,32,33,34,35,36], solution compositions [27], an electric field on as-cast polymer film [37], shear during fabrication [38,39], temperature [40], evaporation rate [41] and humidity [42] have been reported to influence the long-range order and the orientation of self-assembled nanostructures. Solvent evaporation has proven to be a remarkably effective tool for directing the self-assembled nanostructures in the block copolymer thin films. In this approach, two conditions of evaporation were checked: first, normal evaporation in open atmosphere and second, slow evaporation by covering the cast film; fast evaporation was found as the key to forming perpendicular cylinders due to the build-up of a concentration gradient perpendicular to the film surface [41,43]. So far, controlled evaporation using gas flow has not been reported as a tool to control structure formation in order to widen the window of fabrication parameters providing isoporous flat sheet membranes. 

In our recent works on isoporous hollow fiber membranes, with the aim to achieve evaporation-induced self-assembly on the lumen side, we introduced the use of gas flow to trigger and control the microphase separation of block copolymers during SNIPS, we call this modified method “gSNIPS”. By this, the inside-out integral asymmetric and composite isoporous hollow fiber membranes were fabricated via spinning [25] and coating [44] methods, respectively. Yet due to the various structure-controlling factors in the two different hollow fiber membrane fabrication methods, a direct effect of the gas flow rate (*Q_g_*) and the time of gas flow (*t_g_*) on the macromolecular self-assembly and its principles governing the structural formation remains vague. In order to lower the relative humidity (RH) around the as-cast polymer film in the fabrication of NIPS membranes, purging nitrogen in the chamber is a common approach; however, we could not find any detailed study about the influence of nitrogen flow on the membrane structure. Thus, a detailed understanding of the gas-flow incorporated membrane formation process would considerably aid in finding the optimal processing parameters.

The goal of the work presented here was to investigate the self-assembly in diblock copolymer solution films upon controlled solvent evaporation (gSNIPS). For the lab-scale hand casting experiments, we designed a casting envelope which allows precise regulation of *Q_g_* and *t_g_*. Herein, the selected evaporation conditions were evaporation in the atmosphere (we call this normal evaporation) or SNIPS and much faster than the normal evaporation. For the fast evaporation conditions, we introduced a gas flow (mainly nitrogen N_2_ or dry gas) during the fabrication of flat sheet membranes which could provide a controllable evaporation rate to control the microphase separation, and thus the structure formation. The influence of the rate of evaporation on isoporous nanostructure formation, orientation of cylindrical pore-forming domains and the progression of these microphase separated block copolymer domains in the films were investigated and compared with those obtained from the conventional fabrication method. The study is supported by reporting the basic yet important observations regarding the influence of normal or gas flow induced evaporation on the amount of pristine solvents, temperature drop on the surface of the polymer solution and compositional changes in the as-cast film with time.

## 2. Materials and Methods

The specific details of the novel gSNIPS process for the preparation of isoporous membranes under controlled casting conditions and their influence on the membrane structure are discussed in the following sections.

### 2.1. Envelope Design

**Figure 1:**
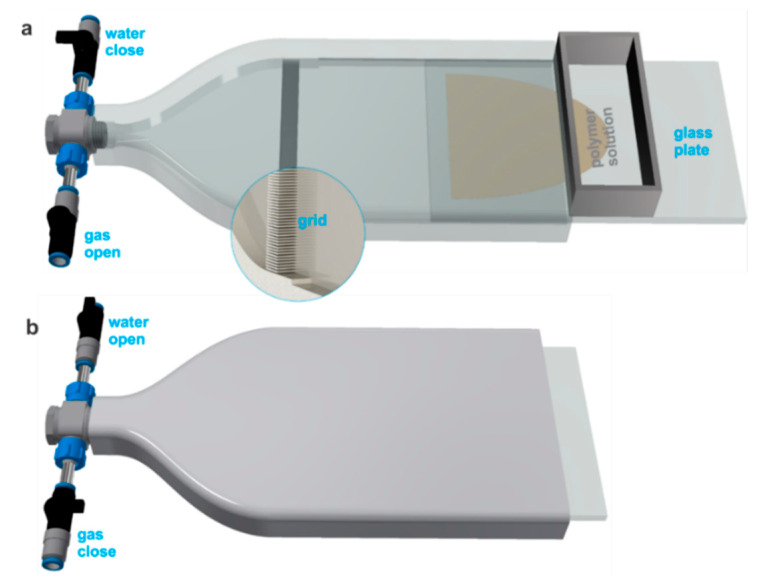
The set-up used for the casting of flat sheet membranes on the glass plate. (**a**) The glass plate was put slightly inside the envelope and the doctor blade was placed on it. To cast the polymer solution, the glass plate was moved inwards while the gas flow was on. (**b**) After a certain *t_g_*, the non-solvent flow was introduced and simultaneously the gas flow was closed. The magnified area shows a grid in the internal view of the casting envelope.

As a prerequisite for performing the lab-scale experiments providing controllable laminar gas flow on the top surface of the as-cast polymer solution, a casting envelope was designed as shown in Figure 1. The dimensions of the inner side of the envelope were decided as 20 × 10 × 2.3 cm^3^ (length × width × height) according to the dimensions of available glass plates, providing a gap of 1.5 mm above the glass plate. The envelope was manufactured by an in-house 3D printer, ProJet MJP 3600 (3D Systems Inc., Rock Hill, CA, USA). To provide a laminar flow on the top surface of an as-cast polymer solution, the distance from the connecting tube to the glass plate was increased by incorporating a triangular shape in the front. This ends at the glass plate with a fine grid of the gap and the material, each having a thickness of 0.5 mm (as shown in the magnified area in Figure 1). Considering the total cross-section at the glass plate (10 × 1.5 cm^2^), *Q_g_* of 1.5 and 4.5 L/min corresponded to the flow velocities of around 0.167 and 0.5 m/s and Reynolds number of around 32 and 97, respectively, providing the laminar flow in the envelope. 

### 2.2. Preparation of Polymer Solutions

The PS-*b*-P4VP diblock copolymers were synthesized via sequential anionic polymerization following a reported protocol, more details are given in the electronic supplimentry information (ESI) [26]. All chemicals used in this study were purchased from Th. Geyer (Renningen, Germany), Sigma-Aldrich (Schnelldorf, Germany), or Merck (Darmstadt, Germany). The solutions were prepared by stirring a specific concentration of block copolymer in a mixture *N,N*-dimethylformamide (DMF) and tetrahydrofuran (THF) in specific weight ratio (wt/wt) for around 48 hours; the solutions were allowed to rest for some hours before the membrane fabrication.

### 2.3. Fabrication of Flat Sheet Membranes

Flat sheet membranes were cast either on the glass plates or on the substrates fixed on the glass plates using a doctor blade of gap height 200 µm. To provide a selected *t_g_* for a certain *Q_g_*, the doctor blade was placed on the glass plate that was moved inwards towards the envelope to cast the film of polymer solution while the gas flow was on. After a certain *t_g_*, the non-solvent (water) flow was introduced using ball valves from the same entrance as the gas flow. To control the *Q_g_*, a mass flow controller (Bronkhorst 5.0 L/min N_2_; accuracy of <1% of the set point) was used, and for the water flow a variable area flowmeter (Krohne DK 800; accuracy of <4% of the set point) was used. The membranes were then washed and kept in DI water and dried afterward. During all the membrane fabrications, the relative humidity was below 40% and the ambient temperature was in the range of 19–21 °C.

### 2.4. Solvent Evaporation and Temperature Drop on the Surface

As it was not possible to measure the evaporation inside the envelope, we imitated the experiments. To check the evaporation effect, 3 mL of solvent was taken in a petri dish of diameter 3 cm. The gas flow was applied near the petri dish having the cross-section of the exit around 5 × 3 cm^2^, which provided similar gas flow velocities for the volumetric gas flow rates as experienced inside the envelope above the glass plate. 

In order to measure the temperature of the surface of evaporating solvents, we used a DualTemp Pro Insertion Infrared thermometer (Dostmann Electronic GmbH). For the measurement of surface temperature, an infrared pyrometer or remote-sensing thermometer was used. The pyrometer has an accuracy of ±1 °C or 1% and a response time of approximately 500 ms, and thus has a very short adaptation period as compared to contact-making methods.

### 2.5. Morphological Investigation

To obtain information about the membrane morphology, scanning electron microscopy (SEM) investigations were carried out on either a LEO Gemini 1550 VP or a Merlin (both from Zeiss, Oberkochen, Germany) at an acceleration voltage of 1–5 kV, respectively. Cross-sections were prepared under cryogenic conditions. All samples were sputtered with ca. 2 nm of platinum as a conductive layer. Secondary electron In-Lens and HE-SE2 detectors were used for imaging the membrane morphology and topography. To obtain the average pore diameter, the SEM micrographs were analyzed by IMS V15Q4 (Imagic Bildverarbeitung AG, Glattbrugg, Switzerland), more details are given in ESI.

## 3. Results and Discussions

### 3.1. Membrane Fabrication under Normal Evaporation Conditions (by SNIPS)

PS-*b*-P4VP diblock copolymer promises the most uniform pores with uniform distribution along with higher surface porosity in the binary solvent mixture of DMF/THF at a ratio of around 1/1 (wt/wt) as compared to other binary or ternary solvent mixtures of DMF, THF, or *1,4*-dioxane [27]. Thus, for this study we selected the homogenous solutions of PS-*b*-P4VP diblock copolymer in DMF/THF 1/1 (wt/wt) unless stated otherwise. The prepared solution remains homogeneous in the steady state. In the conventional fabrication process, a homogenous block copolymer solution is cast either on a glass or non-woven plate and evaporation is allowed under normal environmental conditions. The solvents DMF and THF are selective towards P4VP and PS, respectively. Upon evaporation of the solvent(s), THF tends to evaporate faster because of its higher volatility as compared to DMF, and with increasing *t* the solvent mixture becomes more selective for the P4VP; this leads to the hindered mobility of PS chains while the P4VP blocks remain swollen in DMF. This non-equilibrium microphase separation provides the hexagonally packed uniformly sized P4VP domains in PS matrix for a certain time, at this point, the self-assembly is ceased by immersing the polymer film in a water bath for NIPS [23]. 

**Figure 2:**
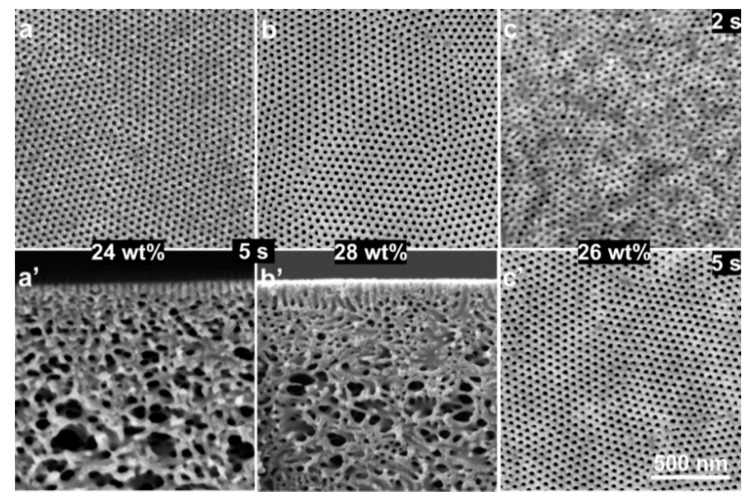
Scanning electron microscopy (SEM) micrographs of the top surfaces (**a**,**b**,**c**,**c’**) and of cross-sections (**a’**,**b’**) of the flat sheets cast using block copolymer solution with 24 (a,a’), 28 (b,b’) and 26 (c,c’) wt% PS-*b*-P4VP_18_^150^ in DMF/THF 1/1 (wt/wt) under normal evaporation conditions.

For the diblock copolymer PS-*b*-P4VP_18_^150^ (where the subscript denotes the amount of the P4VP block in wt% and the superscript denotes the total molecular weight in kg mol^−1^) the optimal solution concentration to obtain an isoporous membrane is 26 wt%, more details about block copolymer characterization are given in ESI. Figure 2 shows that the isoporous structure was obtained for polymer concentrations 24–28 wt% when the RH was below 40%; the selected block copolymer composition was already an optimized one [26]. The analysis of SEM micrographs of isoporous structures does not indicate a significant influence on the average pore diameter (Table 1) while an influence on the membrane substructure is clearly visible. The microphase separation of block copolymer required at least around 5 s for the isoporous structure formation under normal environmental conditions for the polymer concentrations of up to 26 wt% PS-*b*-P4VP_18_^150^, see Figure 2c,c’.

### 3.2. Membrane Fabrication under Controlled Evaporation Conditions (by gSNIPS) and Influence of Block Copolymer Concentration

**Figure 3:**
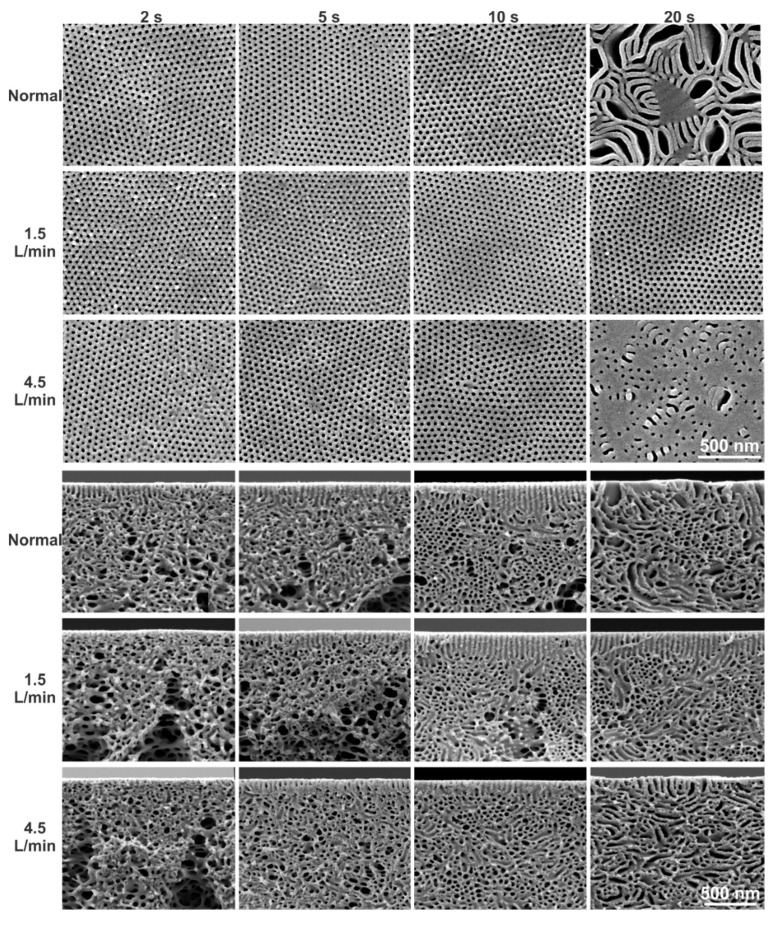
SEM micrographs of the top surfaces and of the cross-sections of the flat sheets cast using block copolymer solution of 28 wt% PS-*b*-P4VP_18_^150^ in DMF/THF 1/1 (wt/wt). As mentioned on the micrographs, the casting was done under normal (SNIPS) and controlled (gSNIPS) evaporation conditions for *Q_g_* of 1.5 and 4.5 L/min (bottom) and for *t_g_* of 2, 5, 10 and 20 s.

In the novel gSNIPS process, the membranes were cast for *Q_g_* in the range of 1.5–6.0 L/min and for *t_g_* of 2–40 s using PS-*b*-P4VP_18_^150^ diblock copolymer solutions with various concentrations. For *Q_g_* of 1.5 and 4.5 L/min, the isoporous structure could be obtained within 2 s for the block copolymer concentrations of 28 wt% PS-*b*-P4VP_18_^150^, see Figure 3. As it is always desired to reduce the polymer consumption without compromising the membrane quality, the study of this work focused on the influence of fabrication parameters on the comparatively less concentrated solutions.

**Figure 4:**
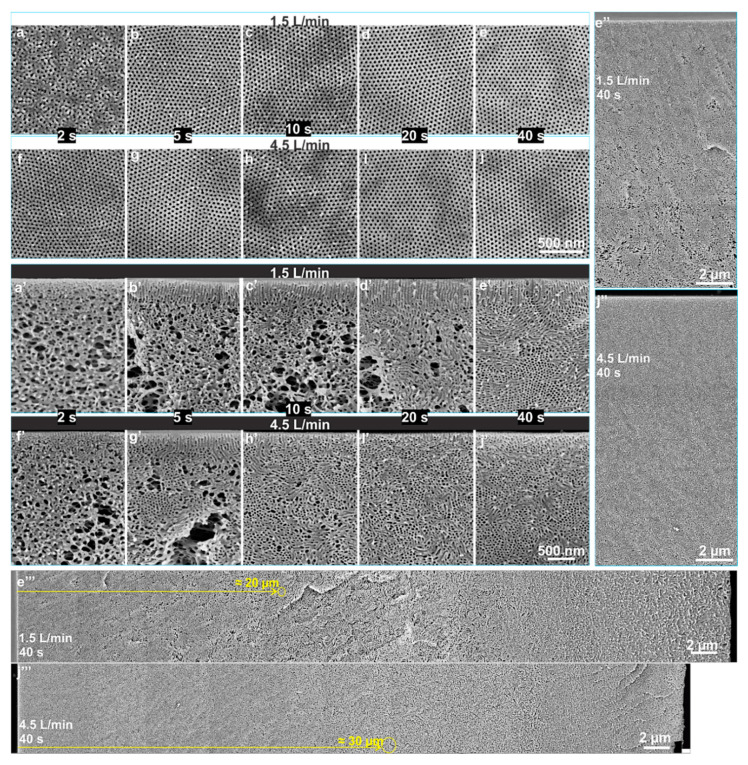
SEM micrographs of the top surfaces (**a**–**j**) and the cross-sections (**a’**–**j’**) of the flat sheets cast using block copolymer solution of 24 wt% PS-*b*-P4VP_18_^150^ in DMF/THF 1/1 (wt/wt). The casting parameters were *Q_g_* of 1.5 (top surface: a–e; cross-section: a’–e’) and 4.5 L/min (top surface: f–j; cross-section: f’–j’) and *t_g_* of 2, 5, 10, 20 and 40 s. All the micrographs, a–j and a’–j’, have the same scale bar. The stacked SEM micrographs in images **e’’**,**e’’’** and **j’’**,**j’’’** show the cross-sectional morphology at different magnifications, for *t_g_* of 40 s and *Q_g_* of 1.5 and 4.5 L/min, respectively.

Figure 4 shows the influence of *Q_g_* (1.5 and 4.5 L/min) and *t_g_* (2–40 s) on the flat sheet membranes cast using a diblock copolymer solution of 24 wt% PS-*b*-P4VP_18_^150^. For *Q_g_* = 4.5 L/min, the isoporous structure formation could be achieved only within *t_g_* = 2 s. However, the reduction in the time required for structure formation from 5 to 2 s in a comparatively dilute solution was not possible for normal evaporation and at low *Q_g_* = 1.5 L/min. The faster development of self-assembled pores on the top surface leads to a faster membrane fabrication.

**Figure 5:**
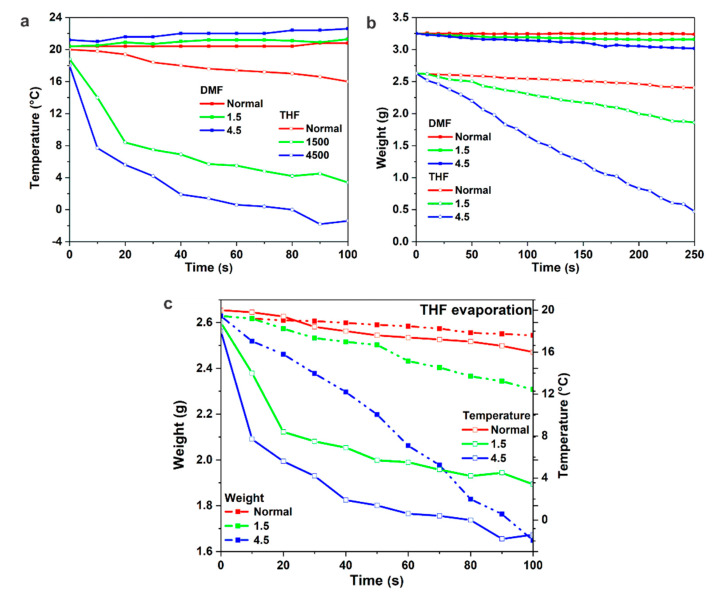
Influence of evaporation conditions (normal evaporation, *Q_g_*: 1.5 and 4.5 L/min) on evaporation (weight, g) (**a**) and temperature (°C) of the top surface (**b**) for solvents DMF and THF. (**c**) Influence of evaporation conditions on the evaporation (weight loss) and temperature drop for THF.

In order to understand the fast self-assembly of macromolecules within 2 s at *Q_g_* = 4.5 L/min, the influence of gas flow was investigated on the evaporation of the pristine solvents THF and DMF. Although evaporation is considered to be a surface phenomenon, the rate of molecular transport across a liquid–vapor boundary is strongly dependent on the coupled fluid dynamics and heat transfer in the bulk fluids. Likewise, the self-assembly is also highly influenced by the temperature of the system especially on the top surface, so the influence of gas flow on the temperature drop on the surface due to evaporation of the solvent was also considered for this study. The simultaneous studies of evaporation rate and temperature drop on the surface was conducted as detailed in Section 2.4. We note that the reported data are the best approximation after several repetitions as the weighing balance kept fluctuating due to the disturbances caused by the gas flow. The evaporation curves in Figure 5a show a strong influence of *Q_g_* on the evaporation of DMF and THF compared to the normal evaporation, which is much stronger for THF compared to DMF due to the difference in their vapor pressures of 173 mbar and 3.8 mbar at 20 °C, respectively.

In contrast with the normal evaporation, the concentration of the solvent in the surrounding is less likely to increase with time during gas flow, due to the regular removal of the evaporating substance. Owing to no evaporation-equilibrium in the enclosed area, gas flow enhances the diffusion of volatile solvent(s) from the inner levels due to free venting condition and there is less exertion on the surface keeping the molecules from launching themselves, thus it boosts the capacity for the evaporation, see Figure 5a,c. For small *Q_g_* = 1.5 mL/min, the pressure measured at the entrance valve in the envelope was 1.034 bar equal to the atmospheric pressure, while with an increase in *Q_g_* from 1.5 to 4.5 L/min (or gas flow velocities 0.167 and 0.5 m/s, respectively) the pressure increased from 1.034 to 1.037 bar. The pressure was understandably higher at the entrance valve as compared to above the glass plate due to the smaller area. As N_2_ flowed through the envelope, it suffered an additional pressure loss. The pressure loss along the envelope was around 0.02 and 0.06 mbar for *Q_g_* of 1.5 and 4.5 L/min, respectively.

Upon evaporation, the composition of the ternary mixture of block copolymer, DMF and THF changes continuously. The rate of evaporation and solvent selectivity play a key role in controlling the microphase separation by controlling the build-up of concentration gradient/chemical potential perpendicular to the film surface [27,41,43]. Therefore, in Table 2, we tried to map the compositional changes of the as-cast film as a function of time upon solvent evaporation under normal and controlled evaporation. We estimated that the 200 µm thick as-cast film from 1 mL 24 wt% block copolymer solution spreads in the area of ca. 50 cm^2^. At a high *Q_g_,* such as 4.5 L/min, after *t_g_* of 5 s the polymer concentration increases to ca. 25 wt%, this variation is even less within 2 s. While in normal evaporation, the isoporous structure could not be achieved before *t_g_* of 5 s for a polymer concentration <26 wt%. This shows that the self-assembly of block copolymers occurs not only because of the increase in the concentration on the top surface upon evaporation, but also depends on the rate of evaporation caused by *Q_g_*, e.g., from 1.5 to 4.5 L/min, providing a quick influence on the swelling of blocks. Here, we want to mention that in flat sheet membranes the possibility of an increase in evaporation and desired structure formation is significantly higher because more surface molecules per unit of the volume are potentially able to escape as compared to inside the lumen of a hollow fiber membrane; the latter has a smaller accessible enclosed volume [44,45,46].

Faster evaporation could provide faster self-organization of macromolecules on the top surface, however, this does not shorten the path to the desired self-assembly of block copolymers due to the significant difference in the evaporation rate of THF and DMF and their selectivity to different blocks. Figure 4b–j shows that for *Q_g_* of 1.5 and 4.5 L/min, the self-assembled uniform pores could be held on for up to 40 s without showing a significant difference in the pore size, see Table 1. This wide window of *t_g_* around 40 s has not been reported yet for isoporous membranes via SNIPS method. While in 40 s, due to the evaporation of THF, the polymer concentration increases from 24 wt% to ca. 36 wt% and the relative amount of DMF increases. As DMF is more selective for the P4VP domains this possibly would lead to the highly swollen cylinders parallel to the surface. A similar phenomenon was also discussed in the simulation study on self-assembly in block copolymer thin films upon solvent evaporation, where hexagonally packed short perpendicular cylinders formed in the earlier stage of the solvent evaporation may remain after solvent removal (dry film) when either the solvent selectivity for the majority block is strong or the solvent evaporation rate is fast [47]. This brings us to consider other parameters influencing the self-assembly of block copolymers in this new set-up of gSNIPS.

In the endothermic process of evaporation, heat is absorbed during evaporation and an increase in evaporation lowers the free energy on the surface. The evaporation rate on the vapor–liquid interface increases with the increase of evaporation temperature and evaporation temperature difference and the decrease of vapor pressure [48]. The role of heat transfer due to evaporation is shown in Figure 5b,c, with an increase in *t_g_* a steep decrease in the temperature can be observed in the curves plotted for pristine solvents, which is much steeper for the selected *Q_g_* as compared to the normal evaporation. This sudden loss of entropy of the macromolecules on the surface of the as-cast solution strongly influences the microphase separation and thus the degree of order. This leads to well-developed isoporous structures within 2 s that are not probable in conventional fabrications. With an increase in *t_g_*, although the as-cast film turns DMF rich, the constantly decreasing temperature and increasing concentration tend to hinder the chain mobility on the top surface. While in the case of normal evaporation the decrease in the temperature on the surface is not so fast it can, however, lead to condensation of water, depending on the RH. This condensation on the surface decreases the kinetic energy of the molecules at its surface and lowers the rate of their evaporation. Figure 5a,b shows a slight increase in weight and temperature on the surface, respectively, during evaporation of DMF under normal evaporation and RH ca. 35%, i.e., due to the hygroscopic nature of DMF. In this modified fabrication method by designing the envelope and applying the gas flow, a long-standing challenge of humidity [42,49,50] in the fabrication of isoporous block copolymer membranes has been solved.

Although no significant influence of the evaporation rate and time can be seen in the structure of the top surface (Figure 4a–j), the cross-sectional morphology (Figure 4a’–j’) highlights the influence of the rate and amount of solvent(s)-evaporation. Under the normal and the comparatively faster evaporation conditions at low *Q_g_* (i.e., 1.5 L/min) the pores oriented perpendicular to the direction of the N_2_ flow or the surface. For low *Q_g_* of 1.5 L/min, growth in the length of cylindrical pores can be seen for *t_g_* from 5 to 40 s that can reach up to 700 nm. This controlled and continuous evaporation of solvents develops a stronger gradient for microphase separation compared to the conventional system. The matrix-forming blocks immobilize while the increased swelling of pore-forming domains in DMF directs the self-assembly of block copolymers to achieve the desired perpendicular orientation of pores, offering good control over self-organization of macromolecules. The increase in rate and time of evaporation moves the segregation of microdomains in a certain depth of the substructure. The cylindrical progression of pores in a micellar solution having a spherical initial state is the result of coalescence of spheres layer by layer during solvent evaporation [39]. This phenomenon can also be facilitated by increasing the segregation strength, e.g., by selective swelling of pore-forming block by varying solvent(s) [27], by introducing additives to the pore-forming block [29,30,39] or by applying an external field [37]. 

Interestingly, for a higher evaporation rate with *Q_g_* = 4.5 L/min, the perpendicular orientation of cylindrical pores vanishes and a rather spontaneous bicontinuous and random orientation of cylinders can be seen for *t_g_* from 2 to 40 s. The cross-sectional micrographs in Figure 4 show that with increases in the rate and time of evaporation, the self-assembly or microphase separation of block copolymers as hexagonal packing of P4VP domains increases in depth from the top in the cross-section and the substructure gets denser with a decrease in the number of voids, as seen in Figure 4e’’–j’’. Figure 4e’’’–j’’’ show the stacked SEM micrographs representing a complete cross-sectional view of a flat sheet membrane, which were provided at *t_g_* of 40 s and high *Q_g_* of 4.5 L/min. This rather symmetric substructure development is due to hindered chain-mobility and thus the growth of microdomains can be confirmed by checking the structure formation in a less concentrated solution for similar evaporation conditions. 

**Figure 6:**
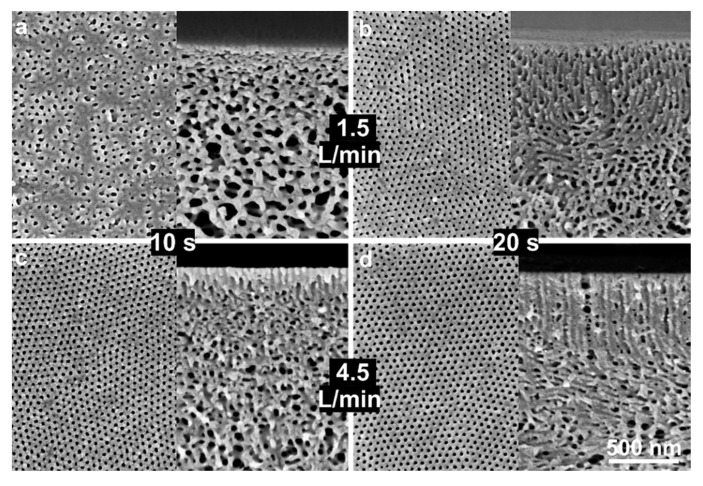
SEM micrographs of the top surfaces and the surfaces of cross-sections of the flat sheets cast using block copolymer solution of 20 wt% PS-*b*-P4VP_18_^150^ in DMF/THF 1/1 (wt/wt). Casting parameters were *Q_g_* of 1.5 (top) and 4.5 L/min (bottom) and for *t_g_* of 10 (**a**,**c**) and 20 s (**b**,**d**).

Figure 6 shows the influence of casting parameters of *Q_g_* of 1.5 and 4.5 L/min for *t_g_* of 10 and 20 s for the diblock copolymer solution of 20 wt% PS-*b*-P4VP_18_^150^. The growth of P4VP domains oriented perpendicular to the surface can be observed for both low and fast flow rates. For low *Q_g_* = 1.5 L/min, *t_g_* = 20 s is required to obtain the isoporous structure formation, while for a higher *Q_g_* = 4.5 L/min this is possible within 10 s, the development of the isoporous structure is shown in Figure S3 in ESI. This difference in the growth of cylindrical pores is probably due to the impact of the decrease in the mobility of polymer chains upon fast evaporation, which hinders the growth of the P4VP domains; this hindrance is obviously more in 24 wt% solution. The evaporation of a solvent selective to the matrix-forming blocks hinders the movement of chains and freezes the PS matrix; the freezing of PS matrix offers an isoporous surface on top for a wide range of parameters while significantly different substructures can be observed. So, initially, the self-assembly of block copolymers initiates on the surface but with the passing *t_g_* and increase in the diffusion of solvent molecules towards the surface, the microphase separation occurs in the substructure as well.

**Figure 7:**
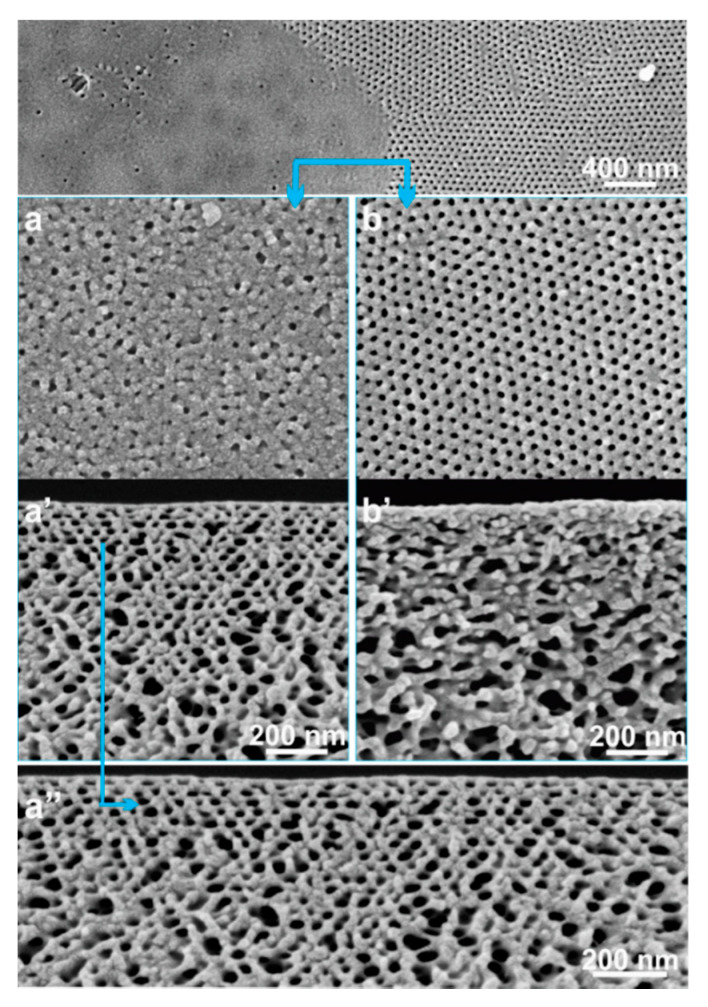
SEM micrographs of the surfaces of top (**a**,**b**) and cross-sections (**a’**,**b’**,**a’’**) of the flat sheet cast using block copolymer solution of 18 wt% PS-*b*-P4VP_18_^150^ in DMF/THF 1/1 (wt/wt). The casting parameters were *Q_g_* = 4.5 L/min and *t_g_* = 10 s.

As discussed, the transition from a disordered/ordered phase in solution to an ordered phase on top of the cast solution depends on the segregation strength between the blocks. Upon evaporation for a longer time that particular concentration can be reached for block copolymers to microphase separate. However, this concept of providing longer evaporation times to obtain the isoporous structure cannot hold under normal environmental conditions due to the condensation of water vapor. For the diblock copolymer PS-*b*-P4VP_18_^150^ in DMF/THF 1/1 (wt/wt), we could obtain the isoporous structure on glass plates with a minimum of 18 wt% of polymer concentration, see Figure 7. For less concentrated solutions, we observed a reduced pore size that is possibly due to the smaller volume of P4VP microdomains. At higher flow rates, we also observed a faster and broader spreading of block copolymer solution during casting, thus providing thinner films as compared to smaller *Q_g_*. Interestingly, as shown in Figure 7, for 18 wt% we observed the influence of gas flow not only as inhomogeneous structures on the top structure but also in the substructure near the top surface. The hexagonal packing of P4VP domains in the direction of flow can be observed in Figure 7a’’. A similar coalescence of spherical micelles into cylinders and their hexagonal packing in the direction of flow was also observed due to shear during isoporous hollow fiber membrane fabrication [39]. This limits the gSNIPS method for solutions having lower polymer concentration.

### 3.3. Influence of Solvents Composition on Structure Formation

It has been reported that the solvent selectivity strongly influences the structure formation not only in solution but also in the fabricated membranes. The difference in the rate of evaporation and together with that variation of temperature of the self-assembling system makes the structure formation more complex. In order to explicitly understand the influence of the rate of evaporation and temperature drop on the self-assembly, the solvent composition of DMF/THF was varied from 1/1 to 7/3 and 3/7 (wt/wt) while keeping the block copolymer concentration constant at 24 wt%. Upon increasing the amount of DMF from 50 wt% to 70 wt% in the solvent mixture, the increased solubility of the minor block causes shrinking of the matrix-forming block, which opposes the desired segregation strength by restricting the movement of the polymeric chains. This solution composition is not capable of providing isoporous structures in a wide range of casting parameters at normal evaporation conditions. However, in the case of gas flow induced evaporation, self-assembly could be observed but not as the desired well-developed isoporous structure formation. Thus, in the case of weakly segregated polymer solutions, the gSNIPS method tends to provide better control on self-assembly due to sudden temperature drop induced segregation and immobilizing the structure. 

**Figure 8:**
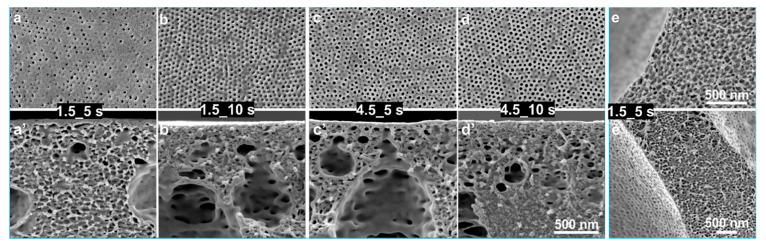
SEM micrographs of the top surfaces (**a**–**d**) and the surfaces of cross-sections (**a’**–**d’**) of the flat sheets cast using block copolymer solution of 24 wt% PS-*b*-P4VP_18_^150^ in DMF/THF 3/7 (wt/wt). As mentioned on the micrographs, the casting parameters were *Q_g_* of 1.5 and 4.5 L/min for *t_g_* of 10 and 20 s. (e,e’) Cross-sectional micrographs near the macrovoids in the substructure.

In the case of increased amount of THF to 70 wt% in the solvent mixture, the self-assembly at normal evaporation does not show a significant influence. While with the gas flow, pits (closed pores) were formed on the surface and the cross-section near the top surface was almost dense except the macrovoids, see Figure 8. The dense substructure is a result of the fast evaporation of THF, which is selective for the matrix-forming block. However, in a certain depth in the cross-sectional micrographs, a packing of spherical micelles can be seen not only as the self-assembled microdomains in the macrovoids but also in their surroundings (Figure 8e,e’). This is due to the increased micellization of block copolymer solution i.e., due to the increased volume of swollen PS domains upon increase in THF. In addition, with increasing the THF content the solution viscosity increases due to the limited solubility of the P4VP segments [27].

### 3.4. Influence of Block Copolymer on Structure Formation

**Figure 9:**
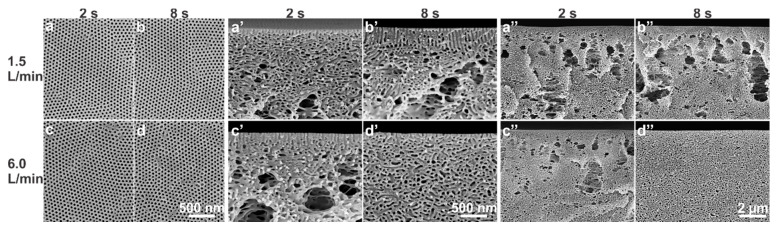
SEM micrographs of the top surfaces (**a**–**d**) and the surfaces of cross-sections (**a’**–**d’**, **a’’**–**d’’**) of the flat sheets cast using block copolymer solution of 27 wt% PS-*b*-P4VP_17_^139^ in DMF/THF 1/1 (wt/wt). As mentioned on the micrographs, the casting parameters were *Q_g_* of 1.5 and 6.0 L/min for *t_g_* of 2 and 8 s.

In order to confirm the above-mentioned results, the experiments were repeated again using two more different block copolymers, PS-*b*-P4VP_17_^139^ and PS-*b*-P4VP_19_^170^, while keeping the solvent composition similar as DMF/THF 1/1 (wt/wt). Since PS-*b*-P4VP_17_^139^ has a similar composition and molecular weight, a similar influence of N_2_ flow on structure formation on the top surface and substructure was observed, see Figure 9. 

**Figure 10:**
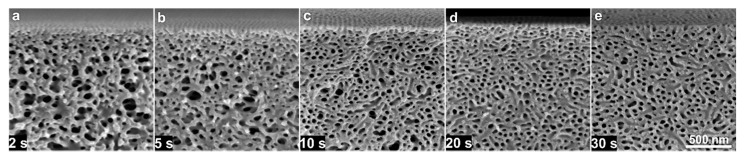
(**a**–**e**) SEM micrographs of the surfaces of cross-sections of the flat sheets cast using block copolymer solution of 22 wt% PS-*b*-P4VP_19_^170^ in DMF/THF 1/1 (wt/wt). The casting parameters were *Q_g_* = 2.0 L/min for *t_g_* of 2–30 s.

With an increase in molecular weight and a rather similar amount of P4VP, the block copolymer PS-*b*-P4VP_19_^170^ shows a difference in the growth and so on the length of cylinders. Figure 10 shows that the isoporous surface could be obtained for a wide range of fabrication parameters, however, in the substructure only densification is observable. The block copolymers microphase separate on the surface but the growth of cylindrical pores in the perpendicular direction is hindered which is possibly due to the reduced mobility in longer chains. Thermodynamically, the higher molecular weight block copolymers tend to microphase separate at lower concentration as compared to block copolymers with the same composition but lower molecular weights. However, the larger fraction of P4VP lowers the mobility of the blocks, thus, hinders the microphase separation and the directional progression of self-assembled microdomains remains restricted [26].

## 4. Conclusions

In conclusion, we introduced a modification in traditional flat sheet casting process by incorporating controlled gas flow in the set-up. For the investigated system, the gas flow induced segregation of block copolymer microdomains, in gSNIPS process, fastens the structure formation within 2 s. The hexagonally packed self-assembled uniform pores can be stabilized for a much longer time (2–40 s) than in the conventional method (5–15 s) without showing a significant difference in the pore size at a certain concentration of the casting solution. 

For the block copolymer used, PS-*b*-P4VP_18_^150^, under normal evaporation the isoporous structure could be achieved for 24–28 wt% concentration while with gas flow this become possible in a much larger concentration window of 10 wt% (18–28 wt%) at one set of casting parameters: *Q_g_* = 4.5 L/min and *t_g_* = 10 s. The stability of self-assembled structures in this modified fabrication method is due to the hindered mobility of polymer chains on the top surface caused by fast solvent evaporation and steep temperature drop. This variability will offer a favorable effect on the production and optimization of materials. Additionally, the modified process solves the issues related to high relative humidity, which is faced in the conventional fabrication method. This study will be helpful to understand the formation of the evaporation-induced self-assembled structures in compact geometries and will ease the understanding of the still complex set of parameters, also for other non-solvent induced phase separation membranes.

The isoporous membranes fabricated by gSNIPS show a strong effect on the membrane substructure while keeping the pore size almost constant. It will be interesting to study the performance of these membranes; the symmetric substructure might reduce the permeability but might increase the durability or mechanical stability during filtrations. 

## Figures and Tables

**Table 1:** Influence of evaporation conditions and solution compositions on the average pore diameter in isoporous flat sheets. The pixel size of the SEM micrographs is 2.23 nm.

Polymer Solution (in DMF/THF 1/1)	*Q_g_*	Average Pore Dimeter (nm)				
*t_g_*		2 s	5 s	10 s	20 s	40 s
18 wt% PS-*b*-P4VP_18_^150^	4.5 L/min			17.0 ± 2.8		
20 wt% PS-*b*-P4VP_18_^150^	1.5 L/min				18.5 ± 2.7	
	4.5 L/min			15.9 ± 3.1	19.7 ± 2.7	
24 wt% PS-*b*-P4VP_18_^150^	Normal		23.0 ± 2.0	22.7 ± 2.4		
	1.5 L/min		23.1 ± 2.2	22.9 ± 2.4	19.7 ± 2.4	19.3 ± 2.5
	4.5 L/min	20.5 ± 2.4	23.3 ± 2.4	23.2 ± 2.7	19.8 ± 2.5	21.5 ± 2.4
28 wt% PS-*b*-P4VP_18_^150^	Normal	20.5 ± 2.4	20.8 ± 2.2	23.3 ± 2.5	22.4 ± 2.6	
	1.5 L/min	18.0 ± 2.7	19.1 ± 2.6	20.8 ± 1.7	22.5 ± 2.0	
	4.5 L/min	17.1 ± 3.1	21.6 ± 2.6	22.6 ± 2.1		

**Table 2:** Influence of evaporation conditions on the solution composition in an as-cast flat sheet film. The evaporating amount is calculated from the curves in Figure 5a. The as-cast film is cast using 1 mL of 24 wt% PS-*b*-P4VP_18_^150^ in DMF/THF 1/1 (wt/wt), which is estimated to spread in 50 cm^2^ area for the thickness of 200 µm.

Time	Evaporation under Environmental Conditions (Normal) and N_2_ Flow								
	Normal			1.5 L/min			4.5 L/min		
	Polymer	DMF	THF	Polymer	DMF	THF	Polymer	DMF	THF
0	24.00	38.00	38.00	24.00	38.00	38.00	24.00	38.00	38.00
5	24.07	38.11	37.81	24.23	38.36	37.41	25.01	39.47	35.52
10	24.14	38.23	37.63	24.47	38.74	36.79	26.11	41.09	32.80
20	24.29	38.46	37.25	24.97	39.53	35.51	28.67	44.81	26.52
30	24.44	38.70	36.86	25.50	40.37	34.14	31.82	49.41	18.76
40	24.59	38.94	36.46	26.06	41.26	32.67	35.82	55.23	8.95

## Supplementary Materials

The following are available online at https://www.mdpi.com/2077-0375/10/5/83/s1, Figure S1: TEM micrograph of the bulk film of the block copolymer PS-*b*-P4VP_18_^150^, Figure S2: Average pore diameter analysis, Figure S3: SEM micrographs of the top surfaces of the flat sheets cast using block copolymer solution of 20 wt% PS-*b*-P4VP_18_^150^ in DMF/THF 1/1 (wt/wt).
