# Supplementary material for: Facilitated Structure Formation in Isoporous Block Copolymer Membranes upon Controlled Evaporation by Gas Flow
Source: Membranes (Basel). 2020 Apr 28;10(5):83. doi: 10.3390/membranes10050083 (PMC7281245; doi:10.3390/membranes10050083)
Supplement: Supplementary file 1 [file membranes-10-00083-s001.pdf]

Supporting Information

Article

Facilitated Structure Formation in Isoporous Block Copolymer Membranes upon Controlled Evaporation by Gas Flow

Kirti Sankhala [1]*, Joachim Koll [1] and Volker Abetz [1,2]*

[1]Helmholtz-Zentrum Geesthacht, Institute of Polymer Research, Max-Planck-Strasse 1, 21502 Geesthacht, Germany
[2] Universität Hamburg, Institute of Physical Chemistry, Martin-Luther-King-Platz 6, 20146 Hamburg, Germany
Emails: kirti.sankhala@hzg.de, Joachim.koll@hzg.de, volker.abetz@hzg.de
* Correspondence: kirti.sankhala@hzg.de, volker.abetz@hzg.de

1. Synthesis and characterization of block copolymer

The asymmetric polystyrene-*b*-poly(4-vinylpyridine) (PS-*b*-P4VP) diblock copolymer was synthesized by sequential anionic polymerization mechanism following a protocol reported before.[1] Tetrahydrofuran (THF, Th. Geyer, Renningen, Germany) was purified by sequential distillation from *sec*-butyl lithium (*sec*-BuLi) (1.4 M in cyclohexane, Sigma-Aldrich, Schnelldorf, Germany) under purified argon atmosphere. Styrene (S) (≥99 wt% with *p-tert*-butylcatechol as stabilizer, Sigma-Aldrich) was purified from basic aluminum oxide (Macherey-Nagel, Duren, Germany) and subsequently distilled from dibutylmagnesium ($MgBu_2$) (1.0 M in heptane, Sigma-Aldrich) under high vacuum. 4-vinylpyridine (4VP) (99 wt% with <1000 ppm *p-tert*-butylcatechol as inhibitor, Sigma-Aldrich) was once distilled under reduced pressure from calcium hydride (CaH_2) and twice from ethylaluminum dichloride ($EtAlCl_2$). The polymerization of styrene was initiated with *sec*-BuLi and allowed to proceed at –78 °C in THF. After 4 h, a small portion of the reaction medium was removed and quenched with degassed methanol to determine the degree of polymerization of the PS block. Afterward, the purified 4VP was added into the solution and stirred for another 16 h at –78 °C. The polymerization was terminated with a mixture of degassed methanol and acetic acid (99%, Sigma-Aldrich). After removal of THF under reduced pressure, the solution was precipitated in Millipore water and the synthesized block copolymer was dried at 40 °C for several days under vacuum to remove the residual solvents and water trace.

The number average molar mass M_n and dispersity index ($Đ$) of the polystyrene precursor and the diblock copolymer were measured by gel permeation chromatography (GPC) (Waters 2410 refractive-index detector, *N,N*-dimethylacetamide as eluent) at 50 °C which was calibrated against PS standards. Using the composition and the molecular weight of the PS precursor the total molecular weight of the block copolymer was calculated. The composition of the block copolymer was determined by ^{1}H-nuclear magnetic resonance spectroscopy (NMR) (300 MHz, in deuterated chloroform) on a Bruker advance 300 NMR spectrometer.

2. Preparation of bulk film and its characterization

For the investigation of the bulk morphology of the PS-*b*-$P4VP_{18}^{150}$ diblock copolymer, thin film was prepared. The solution of 160 mg of block copolymer was prepared by stirring it in 2.5 mL of chloroform for 24 hours and transferred into polytetrafluoroethylene (PTFE) molds

and kept for drying in a hood for two days. In order to equilibrate the sample, *i.e.*, to remove the solvent effect and air bubbles trapped in the samples, the films were further annealed at temperatures below and above the glass transition temperature of both blocks under vacuum. The temperature was gradually increased to 140 °C near the glass transition of P4VP block and finally the sample was annealed for 4 h at 170 °C. The section from the film was used for characterization by transmission electron microscopy (TEM) using a Tecnai G2 F20 (FEI, Eindhoven, The Netherlands) operated at 120 kV in bright-field mode. For this, the film was embedded in epoxy and ultrathin section of ca. 50 nm thickness were obtained by using a Leica Ultramicrotome EM UCT (Leica Microsystems, Wetzlar, Germany) equipped with a diamond knife (Diatome AG, Biel, Switzerland). The ultrathin section was stained with I_2 vapor for 1 h.

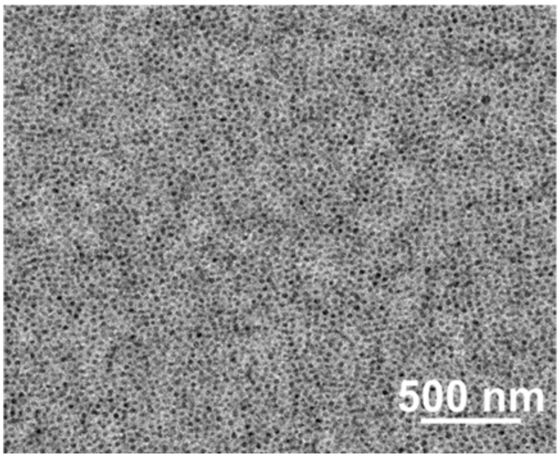

Figure S1. TEM micrograph of the bulk film of the block copolymer PS-*b*-$P4VP_{18}^{150}$.

3. Pore size investigation of SEM micrographs

To obtain the average pore diameter, the SEM micrographs were analysed by IMS V15Q4 (Imagic Bildverarbeitung AG, Glattbrugg, Switzerland). The analysis of a SEM micrograph is shown in Figure S2. The clarity of SEM micrograph is adjusted and binarized in order to find edges of the pores; the green areas correspond to pore area. For structural analysis, the same optimized setting is used for most of the micrographs. In case of defects such as connected pores, corrections were done manually. The average pore diameter is further an average of at least 2 different membrane samples, however, for most of the samples there was not a significant difference in average pore diameters.

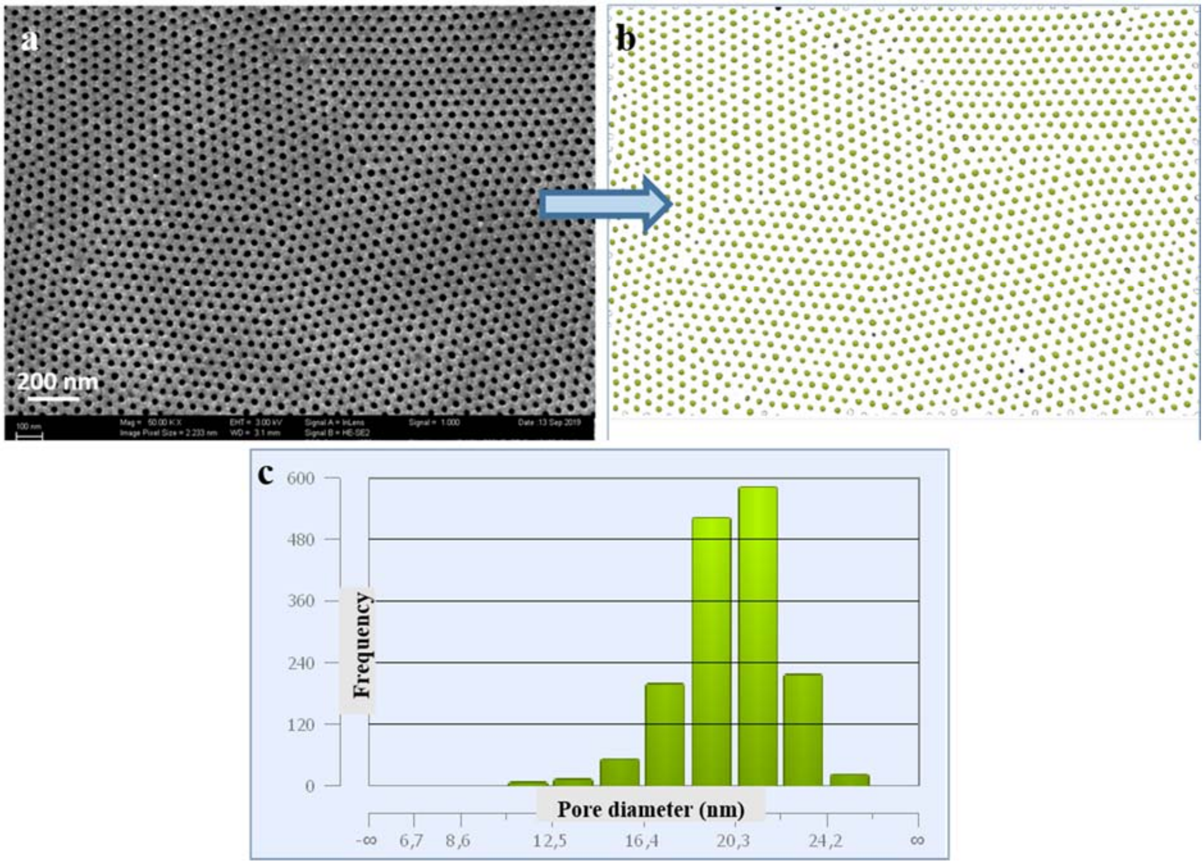

Figure S2. Average pore diameter analysis. a, The SEM micrograph to be analysed. b, The binarized SEM micrograph. c, The plot shows the distribution of pore diameter (nm), where frequency corresponds to the number of pores.

4. Development of the isoporous structure with time

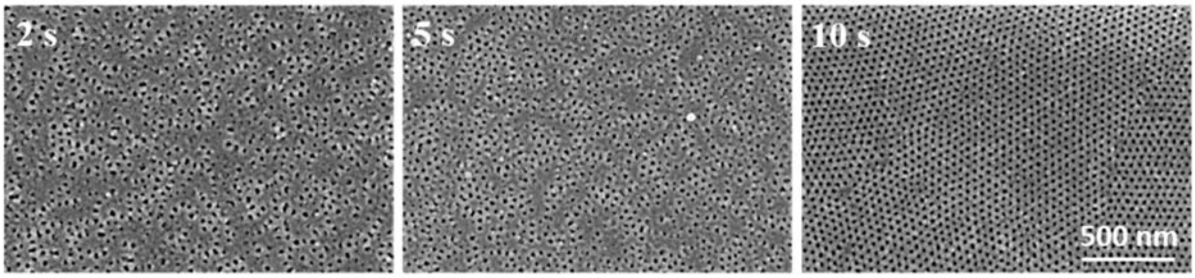

Figure S3. SEM micrographs of the top surfaces of the flat sheets cast using block copolymer solution of 20 wt% PS-*b*-$P4VP_{18}^{150}$ in DMF/THF 1/1 (wt/wt). The casting parameters were Q_g of 4500 mL/min for t_g of 2, 5 and 10 s (as mentioned).

Reference

1. Rangou, S.; Buhr, K.; Filiz, V.; Clodt, J.I.; Lademann, B.; Hahn, J.; Jung, A.; Abetz, V. Self-organized isoporous membranes with tailored pore sizes. *J. Membr. Sci.* **2014**, *451*, 266-275.
